# Core network traffic prediction based on vertical federated learning and split learning

**DOI:** 10.1038/s41598-024-53193-y

**Published:** 2024-02-26

**Authors:** Pengyu Li, Chengwei Guo, Yanxia Xing, Yingji Shi, Lei Feng, Fanqin Zhou

**Affiliations:** 1 6G Research Center, China Telecom Research Institute, Beijing, 102209 China; 2 State Key Laboratory of Networking and Switching Technology, Beijing University of Posts and Telecommunications, Beijing, 100876 China

**Keywords:** Computer science, Information technology

## Abstract

Wireless traffic prediction is vital for intelligent cellular network operations, such as load-aware resource management and predictive control. Traditional centralized training addresses this but poses issues like excessive data transmission, disregarding delays, and user privacy. Traditional federated learning methods can meet the requirement of jointly training models while protecting the privacy of all parties’ data. However, challenges arise when the local data features among participating parties exhibit inconsistency, making the training process difficult to sustain. Our study introduces an innovative framework for wireless traffic prediction based on split learning (SL) and vertical federated learning. Multiple edge clients collaboratively train high-quality prediction models by utilizing diverse traffic data while maintaining the confidentiality of raw data locally. Each participant individually trains dimension-specific prediction models with their respective data, and the outcomes are aggregated through collaboration. A partially global model is formed and shared among clients to address statistical heterogeneity in distributed machine learning. Extensive experiments on real-world datasets demonstrate our method’s superiority over current approaches, showcasing its potential for network traffic prediction and accurate forecasting.

## Introduction

In recent years, network traffic has witnessed a significant surge, propelled by the rapid proliferation of diverse network paradigms such as 5G/6G, Internet of Things (IoT), and Industrial Internet, alongside the increasing popularity of emerging Internet applications like live streaming, video sharing, and virtual reality. This diversification of network services introduces strong randomness, leading to the challenge of providing stable and reliable services. However, adopting traffic prediction can address this issue effectively by capturing the changing trends in user demand. By predicting traffic patterns, networks can proactively deploy communication and computing resources to meet quality of service (QoS) requirements. This predictive capability empowers networks to better adapt to the varying demands of different network services, ultimately providing stable and reliable services.

To achieve satisfactory performance in traffic prediction, researchers have proposed various methods, such as broadly categorized into statistical, machine learning, and deep learning approaches. When modeling network traffic prediction as univariate or multivariate time series, commonly used statistical and machine learning models include Autoregressive Integrated Moving Average (ARIMA)^1^ and Vector Autoregressive (VAR)^2^, etc. This model possesses advantages such as low computational cost and high interpretability. However, it may not be conducive to meeting the requirements of distributed training, and it may encounter challenges in addressing the heterogeneity of data. The majority of deep learning-based network traffic forecasting approaches follow a centralized paradigm. In these scenarios, the forecasting model undergoes training on a central server before deployment. This entails transmitting substantial amounts of raw data from participants to the data center for training a general-purpose prediction model. Such a process can result in excessive data transmission, signaling overhead, potential network congestion, and compromises in payload transmission, raising concerns about participant data privacy. While federated learning addresses data privacy concerns, its performance is often constrained when there is heterogeneity among the training participants’ data.

**Figure 1:**
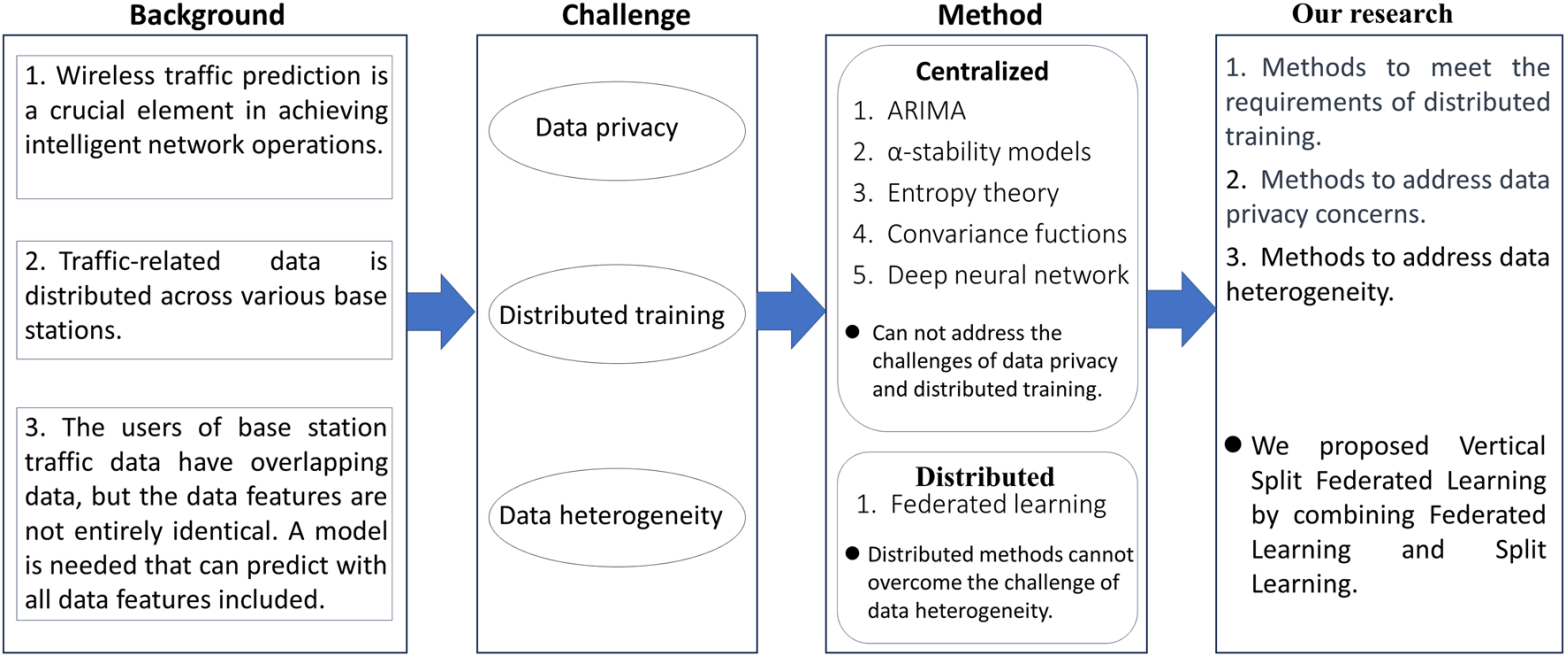
Motivation for the study.

In real-world scenarios, network traffic prediction attracts interest from multiple parties. However, each participant is cautious about sharing their local data due to data privacy concerns. For instance, in the core network context, involved parties may encompass university network management departments, internet service providers, and internet content providers. These entities can be perceived as intelligent agents, equipped with their individual computing and communication infrastructure, capable of participating in traffic prediction tasks. In traffic prediction scenarios where multiple parties demand data privacy, distributed machine learning proves more effective compared to training deep learning models on a single server^3^. Nonetheless, there has been limited exploration of whether this approach yields enhanced performance for network traffic prediction.

The emergence and success of federated learning (FL)^4^ have enabled the resolution of prediction problems while preserving data locality^5^. FL has achieved great success in the medical field. It enables all medical institutions to jointly use disease samples to train disease prediction models with good performance without sharing patient privacy data^6^, and has also made great contributions to the prediction of infection trends during the COVID-19 pandemic^7^. In the FL setting, participants exclusively transmit intermediate gradients or model parameters obtained through local training to a central server, rather than sharing raw data. This approach facilitates model co-training while safeguarding the data privacy of all participating parties. FL holds great potential for application in network traffic^8^; nevertheless, significant research challenges persist and necessitate addressing. User mobility introduces intricate spate-temporal coupling among wireless traffic, presenting difficulties in accurately capturing and modeling it. Moreover, different base stations (BSs) may exhibit distinct traffic patterns, leading to highly heterogeneous traffic data. This heterogeneity poses a considerable challenge for FL to effectively learn and predict traffic data. We have observed that Vertical Federated Learning (VFL) is highly suitable for this scenario, as it adeptly addresses spatial-temporal coupling and handles heterogeneous data, thereby achieving precise network-wide wireless traffic prediction. Additionally, VFL encourages collaborative learning among multiple data owners, all the while protecting the privacy of their respective data. Consequently, VFL emerges as a compelling solution for tackling traffic forecasting problems.

To enhance model training performance while safeguarding user data privacy, a combination of VFL and SL proves beneficial. SL is a distributed learning approach that assigns distinct portions of the model to various devices or participants for computation. In SL, the model’s forward pass primarily occurs on the local devices of the participants, and solely the intermediate representations are transmitted to the central server for further processing. This significantly reduces the amount of sensitive information involved in the transmission process, bolstering privacy protection. By combining VFL and SL, a higher level of privacy protection can be achieved. In this merged approach, participants collaborate using VFL to train the model while employing SL to distribute specific model segments to their respective devices for computation. As a result, sensitive data do not need to be fully exposed to the central server, and only processed intermediate representations are transmitted, further enhancing data privacy protection. This integrated approach effectively balances data privacy and security while facilitating joint learning without compromising performance. Its potential is particularly significant in addressing privacy-sensitive tasks. Therefore, as depicted in Fig. 1, addressing these challenges necessitates the exploration of novel network traffic prediction methods.

Our primary objective is to enhance traffic prediction accuracy in subnets characterized by substantial data heterogeneity through the adoption of VFL and vertical partitioning techniques. By adopting this approach, we can develop high-performing deep-learning models for traffic prediction while ensuring privacy protection, thereby leveraging the advantages of collaborative intelligence. The paper’s primary contributions are as follows:

1. We introduce a distributed machine learning framework tailored for scenarios characterized by diverse data profiles among participants. This framework enables each participant to train directly on their unique dataset through vertical partitioning. Subsequently, the model is aggregated into a comprehensive global model via vertical federated learning. This framework effectively addresses the challenge in federated learning where disparate data characteristics among participants hinder training convergence, making full use of the local data of each participant for training, thus enhancing the efficiency of model training.
2. We propose a novel model training approach for core network traffic prediction by combining federated learning with split learning. Through the application of our method, we effectively address challenges such as data privacy protection, distributed training, and data heterogeneity in the context of core network traffic forecasting.
3. We conducted experiments based on actual network datasets to validate the feasibility of the methods above. The experimental results demonstrate that the proposed framework can significantly enhance traffic prediction efficiency by improving prediction accuracy.

## Related work

In recent years, precise modeling and prediction of network traffic have emerged as crucial elements for various tasks in network communications, garnering significant attention. Network traffic prediction inherently represents a time series forecasting challenge, with solution methods broadly categorized into three main groups: statistical and machine learning methods, deep learning methods, and distributed machine learning methods. The overarching goal of these methods is to offer effective prediction strategies to adeptly handle traffic fluctuations in wireless communications.

Statistical and machine learning methods include parametric techniques that use statistical and probabilistic tools for modeling and predicting wireless traffic. A classical example is Autoregressive Integrated Moving Average (ARIMA)^9^. Researchers have examined ARIMA and its variations to account for self-similarity and burstiness in wireless services. A recent study^10^ decomposed wireless traffic into regular and random components, revealing that ARIMA could predict the regular component but not the stochastic one. In addition to ARIMA, alternative approaches such as $\alpha$-stability models^11^, entropy theory^12^, and covariance functions^13^ have been explored for wireless traffic prediction. These methods aim to better capture the complexity and stochastic nature of wireless traffic. Traditional approaches like ARIMA and $\alpha$-Stable Models have shown drawbacks in adapting to diverse user data features. ARIMA, while straightforward, struggles to capture intricate patterns, making it less effective in dynamic wireless environments. Similarly, $\alpha$-Stable Models face challenges in predicting the self-similar and bursty nature of traffic accurately. Parametric methods such as Entropy Theory and Covariance Functions offer enhanced predictive capabilities but are not immune to limitations. Entropy Theory may fall short in capturing intricate traffic patterns, especially when data features vary among users. Covariance Functions, while contributing to a comprehensive understanding, may encounter challenges in achieving high precision in the presence of diverse data features.

**Table 1:** Methods for numerical prediction and improving prediction performance.

Index	Training methods	Optimization objectives	Data feature	Key technology
Ref.[9]	Centralized	Accuracy	Isomorphism	ARIMA
Ref.[10]	Centralized	Accuracy	Isomorphism	ARIMA
Ref.[11]	Centralized	Accuracy	Isomorphism	ARIMA and SVR
Ref.[15]	Centralized	Accuracy	Isomorphism	LSTM and Lasso
Ref.[16]	Centralized	Accuracy	Isomorphism	Embedding technique and LSTM
Ref.[17]	Distributed	Accuracy	Isomorphism	Federated learning
Ref.[18]	Centralized	Accuracy	Heterogeneity	Metapath and LSTM
Ref.[19]	Centralized	Privacy and accuracy	Isomorphism	Graph convolutional neural network
Ref.[20]	Centralized	Accuracy	Heterogeneity	Locality-sensitive hashing and LSTM
Ref.[21]	Centralized	Accuracy	Isomorphism	Graph convolution network
Proposed	Distributed	Accuracy	Heterogeneity	Vertical federated learning and split learning

In recent years, deep neural network-based approaches have gained momentum. For instance, a wireless mesh network prediction method based on deep belief networks was proposed in a study^14^. Another study^15^ introduced a hybrid deep learning framework that simultaneously captures spatiotemporal dependencies among different cells by combining autoencoders and long short-term memory networks (LSTM). These research endeavors harness deep learning techniques to deliver more robust and accurate solutions to wireless traffic prediction challenges. Despite their contributions to network traffic prediction, these approaches fall short of fully accounting for distinct regional traffic characteristics and scenarios involving distributed intelligence. In the study^16^, embedded techniques address data sparsity and mitigate inaccurate trust predictions caused by feature information forgetting. The authors use LSTM to demonstrate the establishment of trust over time, significantly improving prediction accuracy. Reference^17^ introduces a real-time control algorithm, optimizing model accuracy with dynamic global aggregation frequency within a fixed resource budget. Metapaths and LSTM are employed to address sparsity in trust relationships^18^. In the study^19^ augmented Intelligence of Things and graph convolution network dynamically represent user information, enhancing the recommendation system. However, our paper differs by focusing on resolving data heterogeneity in distributed machine learning through vertical federated learning, complementing challenges not addressed in other papers. LSTM enhances predictive performance in recommendation systems^20^. Our approach differs, This paper concentrates on leveraging distributed machine learning to handle data feature heterogeneity, effectively complementing other works addressing diverse user data heterogeneity.

While LSTM excels in centralized training, our paper emphasizes that the mentioned vertical federated learning approach performs better in distributed scenarios with data heterogeneity. In the study^21^, a method for safeguarding user privacy in recommendation systems is proposed, differing from our paper’s focus. In distributed scenarios, our paper utilizes the differential privacy algorithm, which consistently demonstrates excellent performance. Centralized deep neural network-based approaches, while powerful in centralized scenarios, pose privacy concerns and scalability issues in collaborative training settings with multiple users. Privacy-preserving solutions, therefore, become crucial in the collaborative training landscape. Distributed machine learning methods, such as FL^4^, address the needs of user data privacy and distributed training. However, they may not perform optimally under conditions of data heterogeneity. As depicted in Table 1, we have summarized the optimization objectives, key techniques employed, training modes, and characteristics of training data for various referenced methods. Through comprehensive research, it is evident that only our approach is capable of meeting the collaborative training requirements under distributed conditions, accommodating diverse data features among participating entities.

Unlike the literature mentioned earlier, this article proposes a novel approach that addresses the issue of data heterogeneity through the integration of FL and SL. The adaptability of them to diverse data features among users makes it a significant advancement, offering both robustness and privacy in the collaborative training paradigm. It not only addresses collaborative training challenges effectively but also ensures user data privacy through advanced techniques like differential privacy and homomorphic encryption. This innovative approach contributes to the evolving landscape of wireless traffic prediction, promising more accurate and secure predictions in multi-user scenarios.

## Problem formulation

### The core network base station traffic prediction mechanism

Assume that there are *K* data holders collaborating to train a machine learning model.They hold the local privacy data $\left\{ D_1, \ldots , D_k\right\}$. $D=\bigcup _{i = 1}^KD_i$denotes the data that all can be aligned. The feature space is represented as *X*, The label space is expressed as *Y*, and the sample ID space is represented as *I*. $D_k = (X_k,Y_k,I_k)$. The VFL system assumes *N* alignable samples *D*, $D = \left\{ (x_i,y_i)\right\} _{i=1}^N$, training a joint machine learning model, the label information of the *K*th party is $y_i = y_{i,k}$, each feature vector $x_i \in R_{1\times d}$ distributed among *K* participants $D = \left\{ x_{i,k} \in R^{1 \times d_k}\right\} _{k=1}^K$, $d_k$ is the dimension of the data characteristics of the participant with id *k*. The goal is to use dataset *D* to collaboratively train machine learning models while preserving the privacy of local data and models.

A subnet may consist of multiple base stations, each storing local traffic data, typically including call traffic, SMS traffic, and network traffic. User traffic in a region often exhibits regular patterns, prompting us to utilize historical traffic information for predicting future traffic usage.

The user overlap among these base stations is high; however, each base station only possesses a subset of the data related to user traffic information features. For instance, certain base stations may only have user SMS traffic data, while others might solely have user network traffic data. With the application of our method, we can develop a model capable of predicting the complete traffic features of users.

Given K base stations, each base station has its own local network traffic data, denoted as $d_k = \left\{ d_{k,1}, d_{k,2}, \ldots , d_{k,z}\right\}$, where *Z* is the total number of time intervals. We want to predict future network traffic based on the information of current and historical network traffic. Assuming that $d_{k,z}$ is the target traffic we need to predict, the wireless traffic prediction problem can be expressed in the following form: $d_{k,z} = f\left( \Theta ; d_{k,1}, d_{k,2}, \ldots , d_{k,z-1}\right)$, where *f* is a function, $\Theta$ are the parameters of the model. This equation represents the target flow $d_{k,z}$ based on traffic data from past time intervals $d_{k,1}, d_{k,2},\ldots , d_{k,z-1}$ and the model parameters $\Theta$ to predict. The function *f* defines the specific form of the model, which can be a linear function, a nonlinear function, or other complex models. By learning the model parameters, we can predict future wireless traffic based on the existing historical data. For machine learning-based wireless traffic prediction techniques, only part of the historical traffic data is usually used as input features to reduce the complexity. Therefore, based on the traffic data $d_k$, we can use a sliding window scheme to generate a set of input-output pairs $\left\{ x_{i}, y_{i}\right\}$, Among them $x_i$ denotes the historical flow data associated with $y_i$, Specifically, $x_i$ can be expressed as $\left\{ d_{k,1}, d_{k,2},\ldots , d_{k,z}\right\}$. Here, we focus only on the problem of one-step-ahead prediction. We want to use the traffic of the first n weeks of historical data to predict the traffic at hour t at base station k. If we use 1 h as the minimum time interval, then $s_{week}$ = $(24\times 7=168)$, we denote the prediction by $\widehat{d_{k,t}}$, which can be expressed as

1 $$\begin{aligned} \widehat{d_{k,t}}=f\left( \Theta ; d_{k,\left( t-s_{\text{ week } } \times 1\right) }, d_{k,\left( t-s_{\text{ week } } \times 2\right) }, \ldots , d_{k,\left( t-s_{\text{ week } } \times (z-1)\right) }\right) . \end{aligned}$$

### Objective function formulation

The loss function is defined as follows

2 $$\begin{aligned} \min _{\Theta } l(\Theta ; \textrm{D}) \triangleq \frac{1}{N} \sum _{i=1}^N f\left( \Theta ; x_i, y_i\right) +\lambda \sum _{k=1}^K \gamma (\Theta ). \end{aligned}$$

$\Theta$ is used to represent the shared machine learning model. We can decompose the global model $\Theta$ into local models $\vartheta _k$ parameterized by $\theta _k$, $k\in \left\{ 1, \ldots , K\right\}$, These individual models act only locally, and the global model is represented as $F_k$ with $\Psi _K$ as parameters. Only the *K* participant, known as the active party, can hold. $\gamma (\Theta )$ denotes the loss function and the regularizer. The loss function can be redefined as

3 $$\begin{aligned} \min _{\Theta }f\left( \Theta ; x_i, y_i\right) =\min _{\Theta , \Psi _K} L\left( F_K\left( \Psi _K; \vartheta _1\left( x_{i, 1}, \theta _1\right) , \ldots , \vartheta _K\left( x_{i, K}, \theta _K\right) \right) , y_i, K\right) . \end{aligned}$$

Global model $F_k$ can be the one that needs to be updated using the backpropagation method. The VFL scene is consistent with split neural networks (splitNN), where the whole model is divided vertically into different parts.

In our problem, the data features of *K* base stations are different, and our objective is to enable each base station to utilize its data effectively to minimize the test error. This goal can be reformulated as the minimization of the weighted sum of prediction errors across all *K* base stations. Therefore, we can achieve this by solving the parameter $\Theta$. Through the machine learning approach, we can use the training dataset to fit the model and find the parameter values that minimize the prediction error. Specifically, we can use the input-output pairs $\left\{ x_i,y_i\right\}$ in the training dataset to train the model. By tuning the parameters $\Theta$, we enable the model to obtain the best prediction performance on the training data. Usually, this can be achieved by minimizing the loss function of the prediction error. Once we have finished training the model, we can use the parameter $\Theta$ to make predictions. For a given new input feature, we can use the model and the parameter $\Theta$ to compute the corresponding prediction value. By making predictions on all *K* base stations and comparing the predicted values with the true values, we can evaluate the performance of the model and make further improvements. Thus, by solving for the parameter $\Theta$, we can achieve the goal of minimizing the prediction error at all base stations and improving the accuracy of network traffic prediction.

**Figure 2:**
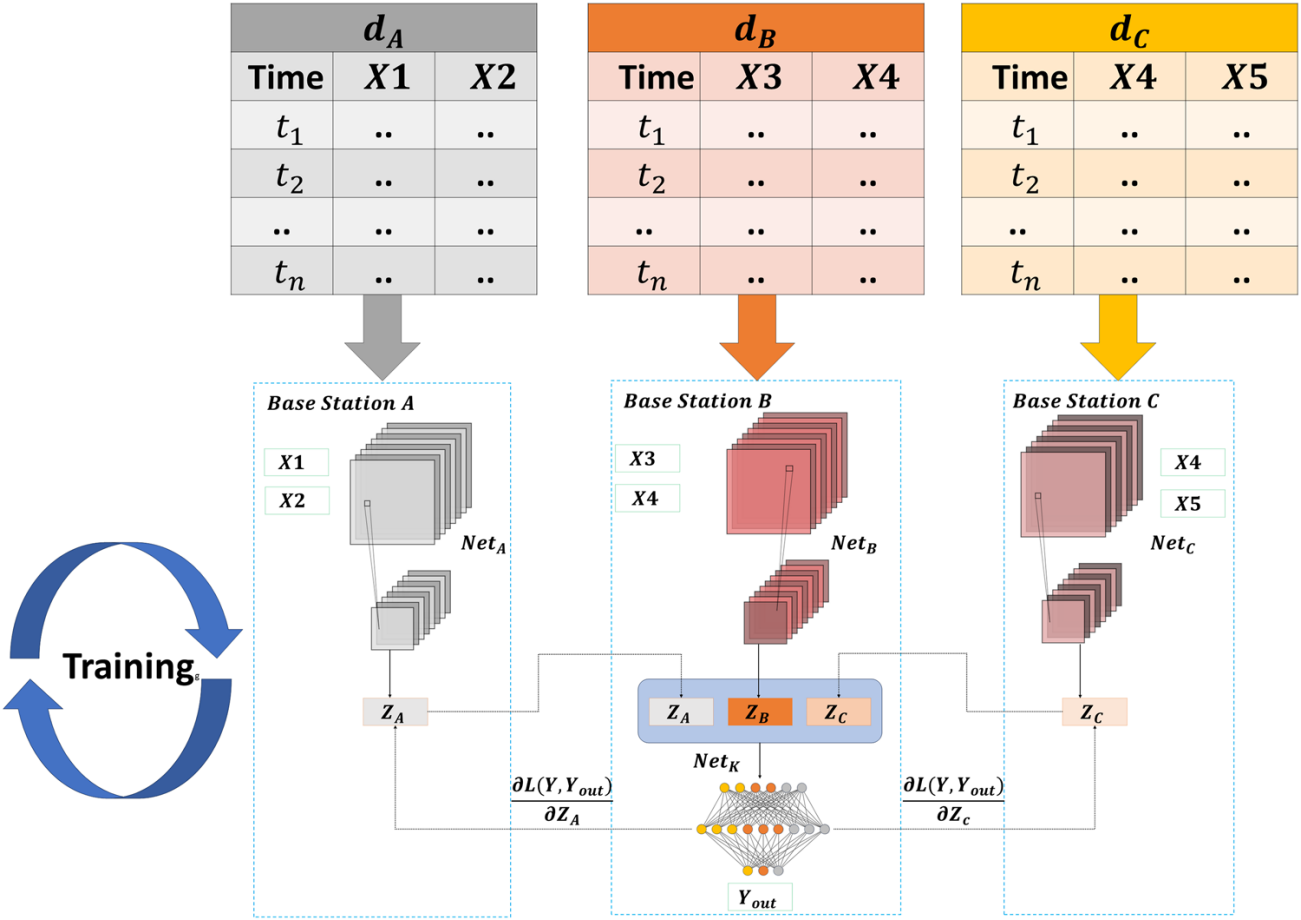
Vertical federated split neural network scheme.

### Overview of the training process

As shown in Fig. 2, Base Station *A*, Base Station *B*, and Base Station *C* each possess a local neural network model: $Net_A$, $Net_B$, and $Net_C$, respectively. These models are employed to extract features from the local training data. Subsequently, the feature representations $Z_A$ on Base Station *A* and $Z_C$ on Base Station *C* are transmitted to Base Station *B*, where they are concatenated with $Z_B$ along the feature dimension. The final output $Y_{out}$ is then generated by passing the merged feature output through another neural network model. There are two key differences to consider in this setup. Firstly, the local model output *z* for a single data sample in logistic regression is a scalar, which needs to be summed up for loss computation. On the other hand, the local output *Z* in the neural network is a vector representing the feature representation. Secondly, Base Station *B* needs to construct an additional neural network model to make predictions based on the concatenated features. It should be noted that the overall output of neural networks in VFL differs from that in centralized learning. This discrepancy arises because the neural network is divided into several separate sub-networks.

In this distributed computation architecture, each base station is responsible for computing a fixed portion of the neuron network. $X_1, X_2,\ldots , X_N$ are the local raw data of these clients, and the features of the data they hold determine the part of the local model they need to train, They calculated and obtained the intermediate features result $Z_1, Z_2,\ldots , Z_N$. The computed portion is then passed to the active party. The active party takes this partial result Combines it into a complete vector *Z* and completes the remaining computations on the network. After completing the computations, the active party performs back-propagation and returns the jacobians (gradients) to the respective station. The stations can then perform their individual back-propagation steps using the received jacobians to update their local model parameters accordingly.

This architecture allows for distributed and collaborative computation, enabling efficient training of complex models in a decentralized manner. By splitting the workload among base stations and utilizing the active party for final computations, the overall training process can be accelerated while preserving privacy and security aspects in certain scenarios.

## Proposed framework

This section provides a specific explanation of the VFL traffic prediction framework used in the problem scenario proposed and demonstrates the complete process of implementing the framework.

Due to the functional differences in urban areas, there are significant differences in base station traffic patterns from region to region, which are necessary to support daily urban operations. In addition, there are differences in users’ mobility and communication behaviors, further increasing the diversity of wireless service patterns. As a result, wireless service data from different base stations are highly heterogeneous, and by nature, they are non-independently and identically distributed (non-iid). Performing federated learning on non-independently and identically distributed data is quite challenging. Traditional federated learning algorithms usually assume that the data are independently and identically distributed, which means that the data from different devices or base stations have similar statistical characteristics. However, these assumptions no longer hold in the face of non-independently homogeneously distributed data, leading to new challenges and difficulties. However, by using our methods and techniques, these challenges can be overcome and accurate and interpretable models can be obtained.

The participating training base stations are divided into active and passive sides, the global model is trainable, and the passive-side local model, after training intermediate results, collaborates with the active-side local model to form the global model *F* and uses the active-side labels for the next training together. The first step for the VFL system to start co-training is to align the data from the base stations. This process, also called entity pairing, uses a technique called private set intersection to find common sample IDs without exposing unaligned datasets, and after alignment, the participants can use the aligned samples to start training the VFL model. Specifically, each base station *k* computes its local model output $H_K = \vartheta _k(x_k,\theta _k)$, on a small batch of samples x, and then sends the local output to the base station of the active party holding the labels.

**Algorithm 1:**
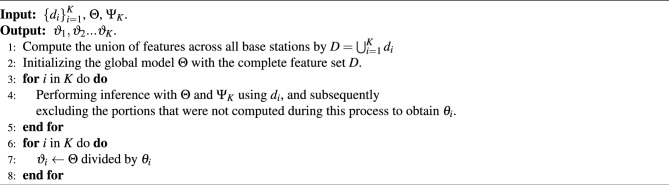
Vertical Split algorithm.

The objective of this scenario is to minimize the error in the inference of the model, Thus, the problem of subnet *k* is formulated as

4 $$\begin{aligned} {{{\rm arg} \min \limits _{\Psi _K}}}\left( L\left( F_K\left( \Psi _K; \vartheta _1\left( x_{i, 1}, \theta _1\right) , \ldots , \vartheta _K\left( x_{i, K}, \theta _K\right) \right) , y_i, K\right) \right) . \end{aligned}$$

The process is described in detail below. Specifically, each party *k* computes its local model output as shown in the following equation

5 $$\begin{aligned} H_K = \vartheta _k(x_k,\theta _k), \end{aligned}$$

where $H_K$ represents an intermediate calculation result. Each participating entity will utilize Eq. (5) to compute over a mini-batch of samples *x* and send the final result $H_K$ to the active party,

6 $$\begin{aligned} \Psi _K^{j+1}=\Psi _K^j-\eta _1 \frac{\partial l}{\partial \Psi _K}. \end{aligned}$$

With all the $\left\{ H_k\right\} _{k=1}^K$, the active party computes the training loss following Eq. (4). Then, the active party computes the gradients $\frac{\partial l}{\partial \Psi _K}$ of its global module and updates its global module using $\frac{\partial l}{\partial \Psi _K}$ as Eq. (6).

7 $$\begin{aligned} \nabla _{\theta _k} l=\frac{\partial l}{\partial \theta _K}=\sum _i \frac{\partial l}{\partial H_{i, k}} \frac{\partial H_{i, k}}{\partial \theta _k}. \end{aligned}$$

Next, the active party computes the gradients $\frac{\partial l}{\partial H_{k}}$ for each party and transmits them back. Finally, each party *k* computes the gradient of its local model $\theta _k$ as Eq. (7). In (7), the chain rule was applied, where the subscript *i* denotes the index utilized in the chain rule for differentiation.

8 $$\begin{aligned} \theta _k^{j+1}=\theta _k^j-\eta _2 \nabla _{\theta _k} l. \end{aligned}$$

Through the VFL training process, we eventually get the parameters $\theta _1,\theta _2,\ldots ,\theta _K$ for the local model and $\Psi _K$ for the global model by Algorithm 1 and get the value of them through a certain number of rounds of iterations. First, we have to set the learning rate $\eta _2$ of the local model and $\eta _1$ of the global model. We may set the participant with the label *K* as the active party holding the label, and for the participants $1,2,3, \ldots , K$ we initialize their model parameters, $\theta _1,\theta _2,\ldots , \theta _K,\Psi _K$. Entering the iterative training process, in each training round, as shown in Algorithm 2, for each base station $k (k=1,2,3, \ldots , K)$, a random sample set $x (x \in D)$ is used for training. First, each participant *k* computes the local model output (4) and then sends the result $H_K$ to the active party *K*. After obtaining the intermediate result for each participant, the active party *K* uses the stochastic gradient descent method to update the global model with (5), and subsequently, the active party *K* computes $\frac{\partial l}{\partial H_{k}}$, and sends it to the other base stations. After receiving the information from the active party, the other participants first calculate (6), and then perform the update of the local model (8). Differential privacy techniques can be used when sending messages.

We employ two evaluation metrics, namely Mean Absolute Error (MAE) and Root Mean Squared Error (RMSE), to assess the effectiveness of the aforementioned method.

MAE is the most common regression metric. Its calculation formula is

9 $$\begin{aligned} \textrm{MAE}=\frac{\sum _{i=1}^n\left| {\widehat{y}}_i-y_i\right| }{n}, \end{aligned}$$

where ${\widehat{y}}_i$ is the predictive value and $y_i$ is the actual value.

RMSE is extended by MAE. It amplifies the error value, and its calculation formula is

10 $$\begin{aligned} \textrm{RMSE}=\sqrt{\frac{\sum _{i=1}^n\left( {\widehat{y}}_i-y_i\right) ^2}{n}}. \end{aligned}$$

**Algorithm 2:**
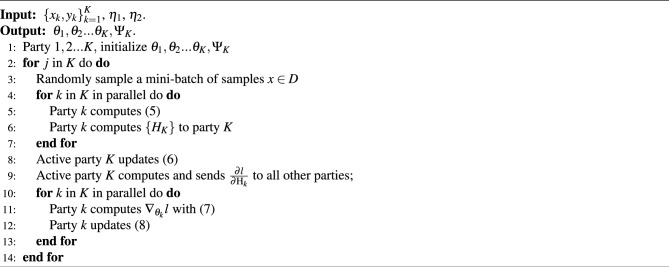
Vertical Split Federated Learning algorithm.

## Methods and results

We used in our experiments the cellular traffic datasets provided by Telecom Italia^22^ The detailed experimental parameter settings are shown in Table 2. These two datasets record the call details of Milan (MI)^23^ in the last two months of 2013. They are among the most commonly used datasets in the field of cellular traffic forecasting^24^. The datasets contain five types of traffic, including SMS input/output, voice call input/output, and Internet services, and are recorded at spatio-temporal granularity. The detailed parameter configurations for our experiments are provided in Table 2. In our experiments, we focus on voice call traffic and Internet service traffic, which are the most common types of cellular traffic in existing networks. Our task is to predict the traffic in week 7 based on the traffic in the first six weeks. We divide the historical traffic data into intervals of the minimum scale of hours and then use the traffic data for each hour of the week to predict traffic data for the corresponding hours in the next week. Specifically, we base our predictions on the traffic data for the 168 h (24 $\times$ 7) per week available in the historical dataset. In addition, we normalize the traffic data so that the traffic within each grid has zero mean and unit variance. By normalizing, we can eliminate scale differences between grids to ensure that the model treats the data fairly across grids. To summarize, we performed preprocessing operations on the dataset, including aggregating statistical intervals to the hour, intercepting data to avoid holiday effects, and normalizing the flow data to ensure that the data have a uniform scale. We compare our proposed traffic prediction framework with four baseline methods as follows:

**Table 2:** Experimental parameter settings.

Parameter name	Parameter values	Parameter meanings
Bs	100	Number of base stations
Frac	0.1	Fraction of clients
Local-epoch	50	The number of local epochs
Local-batch	40	Local batch size
Epsilon	1	Stepsize
Lr	0.01	Learning rate of NN
Opt	Sgd	Optimization techniques
Momentum	0.9	Momentum

1. Lasso: A linear model for regression.
2. LSTM^25^: LSTM exhibits a robust capacity for modeling time series datasets and typically outperforms linear and shallow-learning models in terms of prediction accuracy.
3. Support Vector Regression (SVR)^26^: SVR, a classical machine learning algorithm, has found successful applications in traffic prediction.
4. FedAvg^27^: First introduced in pioneering federated learning research, FedAvg employs weight averaging from local models for aggregation.

To ensure generality and reduce computational complexity, we randomly selected 100 base stations in each dataset and conducted experiments on three types of wireless traffic from these base stations. In the experiments, we used the traffic from the first seven weeks to train the prediction model, while the traffic from the last week was used to test the performance of the model. By randomly selecting 100 base stations, we can reduce the complexity of computation and processing while retaining data diversity. Such a sampling method can represent the characteristics of the entire dataset and provide reliable results in the experiments. The training model uses the first seven weeks of traffic data so that the model can learn the patterns and trends of the historical data. We then use the trained model to make predictions for the last week of traffic to evaluate the performance of the model on future data. With such an experimental design, we can verify the accuracy and reliability of the prediction model and provide meaningful results for further analysis and decision-making. Also, since we randomly selected 100 base stations, our experimental results can be generalized over the entire dataset. We use two evaluation metrics, MAE and MSE, to evaluate the effectiveness of the above method.

**Figure 3:**
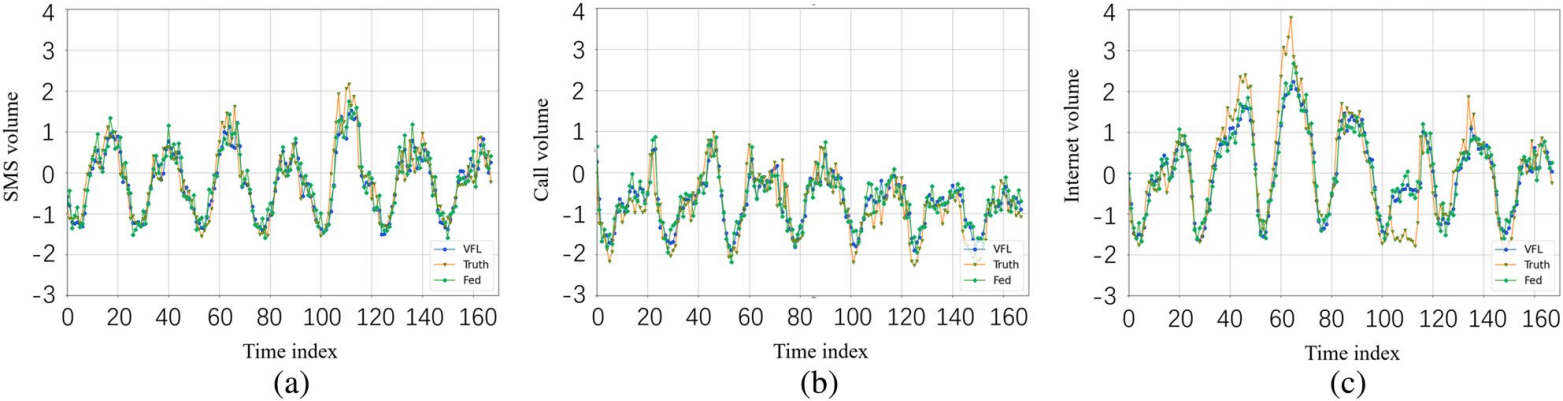
Comparisons between predictions and the real values of Milan datasets.

It is evident from Tables 3 and 4 that our proposed method, VFL, outperforms all the baseline methods across all types of wireless traffic in the Milan datasets and Trento datasets. To further assess the predictive performance of different algorithms, we provide comparisons between the predicted values and the actual values for each algorithm in Fig. 3. In Fig. 3, the results are presented for the Milano dataset. Specifically, the three subfigures of Fig. 3 display the comparisons between the predictions and the ground truth for SMS, Call, and Internet service traffic of randomly selected cells. Here, we select FedAvg as the benchmark for performance comparison since it achieves the best performance among all baseline methods, as shown in Table 3. By analyzing Fig. 3, we can observe that VFL consistently achieves better prediction performance than FedAvg across all three types of wireless traffic. Furthermore, VFL exhibits smaller prediction errors, particularly when dealing with high and unstable traffic volumes.

**Table 3:** Comparison of MSE and MAE prediction performance of different methods on Milano dataset.

	Milano					
Methods	MSE			MAE		
	SMS	Call	Internet	SMS	Call	Internet
SVR	0.5294	0.1211	0.1252	0.3981	0.2134	0.3120
Lasso	0.8411	0.3215	0.4621	0.7214	0.5162	0.6122
LSTM	0.5922	0.1545	0.1874	0.4721	0.3134	0.3122
Fedavg	0.4853	0.1466	0.1168	0.4176	0.2045	0.3109
VFL	$\mathbf {0.3479}$	$\mathbf {0.1023}$	$\mathbf {0.1132}$	$\mathbf {0.3742}$	$\mathbf {0.2001}$	$\mathbf {0.2976}$

The optimal values are in bold.

**Table 4:** Comparison of MSE and MAE prediction performance of different methods on Trento dataset.

	Trento					
Methods	MSE			MAE		
	SMS	Call	Internet	SMS	Call	Internet
SVR	5.3142	1.1823	5.8086	1.1322	0.5721	1.0329
Lasso	4.6123	1.6322	5.6235	1.3221	0.8342	1.5237
LSTM	3.2384	1.2344	4.5723	0.9328	0.5217	1.1356
Fedavg	2.1322	1.4563	4.5232	0.7525	0.5349	1.0348
VFL	$\mathbf {1.8246}$	$\mathbf {1.0023}$	$\mathbf {2.3452}$	$\mathbf {0.6231}$	$\mathbf {0.4012}$	$\mathbf {0.7162}$

The optimal values are in bold.

**Figure 4:**
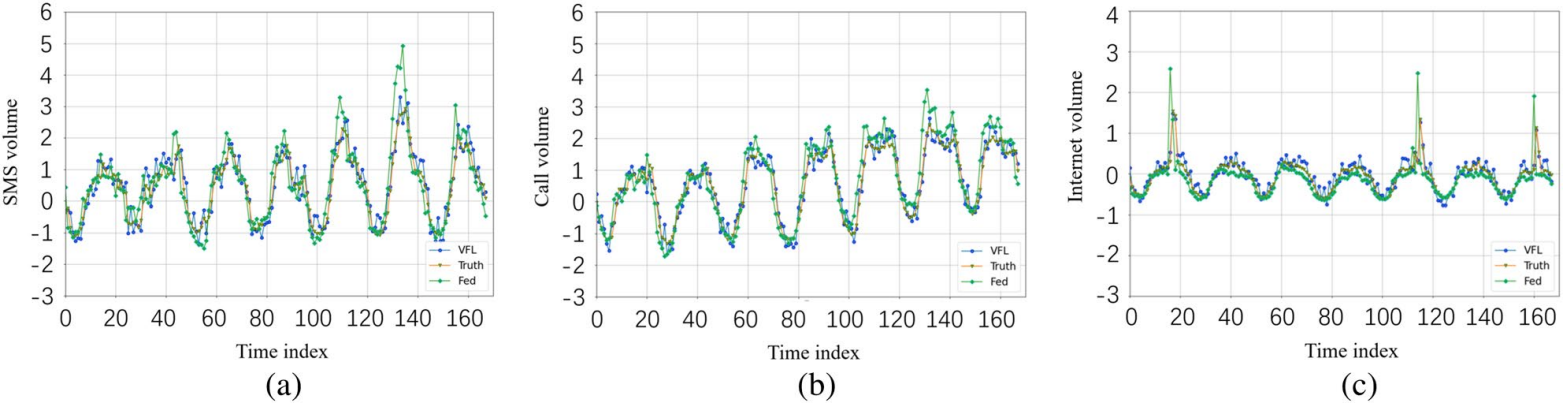
Comparisons between predictions and the real values of Trento datasets.

The results presented in Table 4 and Fig. 4 demonstrate that our method achieves superior prediction performance on the Trento datasets as well. By integrating both VFL and splitNN, our approach effectively captures both spatial and temporal dependencies, leading to improved prediction accuracy. Moreover, our method significantly reduces data heterogeneity compared to traditional FL algorithms, enabling a high generalization capability. It strikes a balance between data from different base stations during training, resulting in more accurate predictions. Compared with fully distributed algorithms that consider only the temporal dependence of network traffic operations (e.g., SVR and LSTM), our approach can capture both spatial and temporal dependence through model fusion, resulting in greater robustness. Compared to traditional FL algorithms, our approach allows the learning process to be tuned for specific cases. In addition, the application of longitudinal federation greatly reduces the impact of heterogeneity of data. As a result, our method has a high generalization capability and can better adapt to the differences and characteristics among different base stations. Our approach is able to strike a balance between capturing the unique characteristics of base station clusters and the macro traffic patterns shared among different clusters. This allows our method to provide more accurate prediction results while balancing the specificity of individual base stations with the shared nature of the overall traffic patterns.

## Conclusion and discussion

In this work, we study the problem of wireless traffic prediction and propose a VFL framework for traffic prediction based on the heterogeneity of base station data characteristics. Dedicated traffic prediction models for subnets with specific characteristics are obtained through VFL. We designed a training architecture combining VFL and splitNN and trained a model through this architecture. Experimental results show that the framework improves the traffic prediction efficiency of the model by solving the problem of different data characteristics between base stations, allowing base stations with different data characteristics to participate in the FL process at the same time. We finally verified the effectiveness and efficiency of VFL on two real-world datasets. However, there are also some shortcomings. On the one hand, predicting future traffic in this framework completely relies on historical traffic, lacking the use of other multidimensional data, such as regional population density and emergency event information. These data are valuable for cellular traffic forecasting, and we will conduct further research in the future.

## Data Availability

All data generated or analyzed during this study are included in this published article (and its Supplementary Information files).
